# The Role of Recombination for the Coevolutionary Dynamics of HIV and the Immune Response

**DOI:** 10.1371/journal.pone.0016052

**Published:** 2011-02-18

**Authors:** Rafal Mostowy, Roger D. Kouyos, David Fouchet, Sebastian Bonhoeffer

**Affiliations:** 1 Institute of Integrative Biology, ETH Zurich, Zurich, Switzerland; 2 Biométrie et Biologie Évolutive, Université de Lyon, Villeurbanne, France; Institut Pasteur, France

## Abstract

The evolutionary implications of recombination in HIV remain not fully understood. A plausible effect could be an enhancement of immune escape from cytotoxic T lymphocytes (CTLs). In order to test this hypothesis, we constructed a population dynamic model of immune escape in HIV and examined the viral-immune dynamics with and without recombination. Our model shows that recombination (i) increases the genetic diversity of the viral population, (ii) accelerates the emergence of escape mutations with and without compensatory mutations, and (iii) accelerates the acquisition of immune escape mutations in the early stage of viral infection. We see a particularly strong impact of recombination in systems with broad, non-immunodominant CTL responses. Overall, our study argues for the importance of recombination in HIV in allowing the virus to adapt to changing selective pressures as imposed by the immune system and shows that the effect of recombination depends on the immunodominance pattern of effector T cell responses.

## Introduction

**Figure 1:**
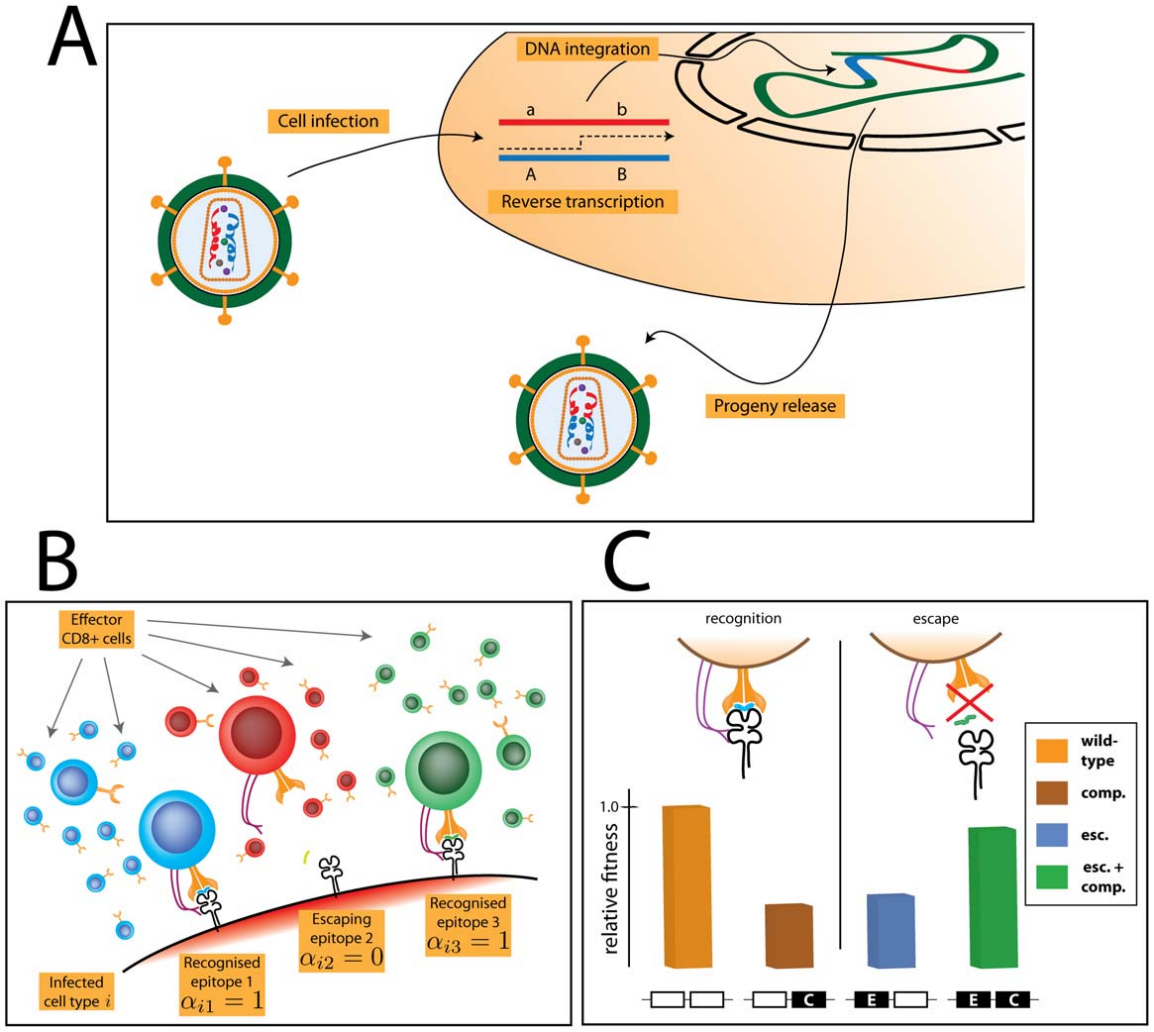
The model of recombination in HIV. Panel A: Recombination in HIV occurs in virions that contain two non-identical copies of RNA. During reverse transcription, one or more crossover events between the two strands can take place and result in recombination. Panel B: Immune escape of the infecting virus is modelled by assuming that 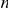 of the epitopes presented on the surface of infected cells are recognised by the CD8+ T cells. The figure shows a situation for 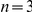 and an infected cell 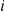, which contains two epitopes recognised by the immune system (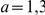) and one epitope that escapes the surveillance of the effector T cells (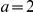). Panel C: Escape from immune recognition comes at a fitness cost for the virus, and thus we assume that an escape mutation impairs the reproductive capacity of the pathogen (blue bar). A compensatory mutation partially restores the fitness cost (green bar). In the absence of the escape mutation, the compensatory mutation not only decreases the replicative capacity of the virus but also does not confer immune-resistance (yellow bar).

Human immunodeficiency virus (HIV) frequently undergoes recombination [1]–[3]. The high recombination rate is the consequence of both a high switching rate of reverse transcriptase between the two viral RNA strands during proviral synthesis [4]–[7] as well as of the large fraction of infected cells that harbour more than one provirus [8]–[10] (see [1] for review). Thus, when two non-identical virions infect the same cell, RNA from two different viruses can be packaged into a new virion, and upon a subsequent infection, a crossover event between the two RNA strands can result in Êrecombination of the genetic material (see Fig. 1A). The evolutionary implications of recombination have been, to some extent, addressed in the context of the evolution of anti-retroviral resistance in HIV [11]–[16]. However, the effect of recombination in HIV on coevolutionary dynamics between the virus and the immune system has, to the best of our knowledge, not been addressed.

HIV is known to evade the immune control, for example by escaping from the recognition by cytotoxic T lymphocytes (CTLs). These CTLs recognize and kill cells that display fragments of viral peptides on their surface. More specifically, proteins produced by the virus inside an infected cell are processed by the host cell machinery into antigenic peptides, and loaded onto MHC class I molecules. The resulting MHC:peptide complexes are then presented on the surface of the cell such that the host's CTLs, which scan the MHC:peptide complexes, can recognize and destroy the infected cells. Viruses can evade the immune response, and studies of HIV-1 and SIV infections have shown selection of CTL escape variants during both primary and chronic infections [17]–[20]. The escape is mediated by the ability of the virus to alter the presented peptide sequences (see Fig. 1B). The mutated proteins then become invisible to the antigen-presenting machinery (e.g. TAP or proteasome), dissociate from the MHC molecule, or become unrecognisable to the effector T cell receptors [21], [22]. On the other hand, escape mutations typically come at a cost to the virus by decreasing the viral replicative capacity [23], [24], although compensatory mutations which partially restore the fitness can emerge [25], [26] (see also Fig. 1C). Thus, HIV can efficiently escape the selective pressure of the immune system.

The immune escape dynamics in HIV can generate an environment which is beneficial for recombination. When the immune system generates immune responses against frequent virus strains, a strong selective pressure arises which leads to the escape of the virus. The immune system in turn produces novel responses that can recognize the escape variants and lead to further escape. The resulting coevolutionary arms race between the virus and the immune system resembles the so-called Red Queen dynamics between hosts and parasites, where recombination has been shown to play an important role [27]–[29]. In such a scenario, parasites adapt to infect frequent host genotypes, which gives rare host genotypes a selective advantage. Under these conditions recombination can give the host an edge in the arms race with the parasite by rapidly generating resistant genotypes. Thus in terms of the classical Red Queen terminology, HIV plays the role of the “host” and the immune system plays the role of the “parasite”. Given the parallel between host-parasite coevolution and virus/immune-system dynamics, it is plausible that recombination may, under appropriate conditions, help the virus to escape from the immune system.

In this study, we examine the role of recombination for immune escape of HIV by using a population dynamic model of HIV. We find that recombination accelerates the evolution of escape in the virus in the early stage of infection. We also find that the CTL hierarchy is of vital importance to viral/immune coevolution as well as viral recombination. In particular, we find that the immunodominance pattern of the CTL response strongly affects the mean number of escapes in the virus as well as the impact of recombination. Furthermore, we determine which parameters increase or decrease the impact of viral recombination on the immune escape, and discuss in which situations genetic shuffling could contribute to the viral immune escape as well as to adaptation of HIV to the host in general.

## Methods

### General construction of the model

Mathematical models have proved an excellent tool for studying complex viral dynamics *in vivo* [30], [31] as well as investigating population genetic and dynamic consequences of recombination [32]. Thus, in order to examine the impact of recombination on the viral immune escape we consider a population dynamic model which is an extension of a standard HIV dynamics model [33]–[35]. The model mimics the interaction of HIV with the immune system by taking into account some basic properties of the coevolutionary dynamics between the virus and the immune system. The model can be expressed by the following set of ordinary differential equations:
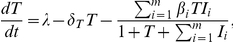


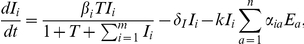


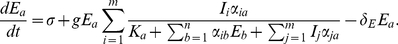
(1)


Three populations of cells are considered: susceptible naive CD4+ T cells (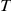), infected CD4+ T cells (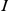), and effector CD8+ T cells or CTLs (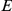). Naive target cells, 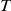, are produced at a constant rate 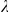, and die at a rate 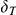. They can become infected by any of 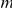 distinct viral strains in the population. A naive T cell infected by a virus strain 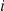 becomes an infected cell of type 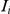. The virus is assumed to be in quasi-steady state [36], which implies that free virus is, to a good approximation, proportional to the infected cells at all times. The transmission rate of an infected CD4+ cell of type 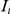 is then defined as 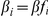, where 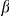 denotes the transmission rate by cells infected with a wild-type strain and 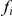 denotes the relative reproductive fitness of the virus strain 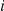 (which implies that 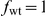).

An infected cell displays 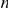 distinct types of MHC:peptide complexes, all of which are recognized at the beginning of infection by effector cells (CTL of type 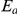 recognizes an epitope 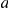). Mutations at these epitopes can lead to escape from recognition by CTLs and here we focus solely on those mutations that result in a complete lack of recognition by the immune system. Whether the clone 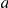 recognizes the viral strain 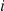 is defined in a binary interaction matrix 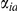, where 1 denotes recognition and 0 denotes escape (see Fig. 1B). We assume that each viral epitope consists of two loci: an escape locus and a compensatory locus. A single mutation in either locus leads to an impairment of the replicative capacity respective to the wild-type (each fitness value is randomly drawn from a uniform distribution between 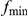 and 1), while a double mutation partially restores it (uniformly drawn from the higher fitness of the two loci and 1; see Fig. 1C). The fitness of each strain 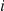, 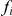, is then the product of individual epitope fitness values 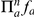.

All infected cells 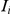 die at a rate 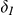 due to reasons other than immune killing (e.g. cytopathicity), and we assume that 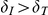. In addition, they are killed by the immune response at a rate 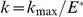 per day, where 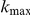 is the maximal death rate of infected cells due to CTL killing and 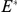 is the number of CTLs in the steady state in the absence of escape (see Table 1). Furthermore, the strength of killing depends on how well the viral strain is recognized by the immune system, and hence is proportional to the number of CTLs specific for the particular strain 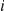, namely 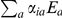. Likewise, the activation and production of the effector sub-population 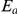 is proportional to the viral sub-population recognized by this clone. The effector cells 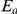 are produced at a constant rate 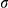, are activated to proliferate at a maximal rate 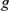, and die at a rate 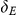.

**Table 1:** Assumed parameter values for HIV dynamics.

Parameter	Assumed value	Name & reference
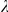	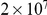 cells/d	Daily production of CD4+ target cells [31], [55]
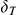	0.01 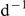	Natural death rate of naive CD4+ target cells [56], [57]
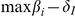	1.5 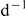	Initial replication rate of the viral population [58]; taken from SIV assays
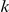	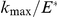 cell 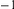 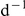 ; 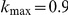 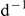 by default	Assumes maximal CTL kill rate at 0.9 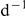 , and otherwise ranged between 0.1 - 10 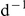 [40], [59], [60]
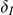	0.1 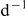	Death rate of virus-infected cells not due to the CTL killing; chosen such that the total HIV death rate is 1 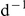 by default [61]
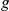	1.0 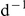	Maximal proliferation rate of CD8+ cells [62]
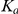	variable; 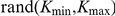 by default	Distribution of dissociation rates; defines the immunodominance pattern of the response
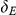	0.01 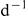	Death rate of CD8+ cells [63], [64]
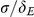	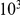 cells	Number of CD8+ cells of type a in the absence of infection; chosen based on the assumption that CD8+ T cell clones increase 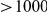 -fold upon stimulation [65]
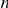	variable, 15 by default	Number of targeted epitopes [66], [67]
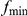	variable, 0 by default	minimal fitness due to single mutation [68]
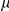	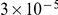	Virus mutation rate per locus [69]
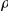	variable; 3 by default	Mean number of crossovers per genome [1], [70]
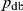	0.1	Percentage of doubly infected cells [9], [71]

The rates of new infections and CTL production are assumed to obey Michaelis-Menten dynamics, which generates a saturation effect in these terms similar to the Michaelis-Menten enzyme-substrate kinetics [34], [37], [38]. By dividing the standard mass-action term by the density of the cells that undergo such kinetics, one generates more realistic and less oscillatory population dynamics of virus/immune-system interactions. The terms describing target cell depletion and the increase of infected cells saturate as a function of the total of naive and infected CD4+ cells, reflecting the T cell density-dependence of the transmission rate 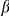 [34]. Likewise, the proliferation via activation of the CTLs saturates as a function of infected cells and effector cells reflecting a non-trivial CTL kinetics *in vivo*, including competition for antigen and saturation in killing [35], [39], [40]. The constant 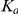 is a dissociation parameter, which is an inverse of the measure of sensitivity (avidity; see below) of the effector T cell clone 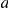: low 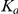 corresponds to a sensitive clone, and high 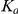 corresponds to an insensitive clone. By varying the distribution of the parameter 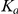, the immunodominance of an effector response is altered. The overview of parameters together with the assumed values is given in Table 1.

### Mutation and recombination

Mutation and recombination are implemented as stochastic events and are executed at discrete time steps during the simulation. When new mutants and recombinants are generated, the ancestral viruses are removed from the population of infected cells, new strains are added, and the set of equations (2) is restructured accordingly. Importantly, both mutation and recombination do not alter the number of infected cells but only the genetic composition of the provirus population.

In order to perform mutation, we approximate the number of CD4+ cells infected with a single virus of type 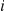 between the time 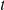 and 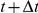 as



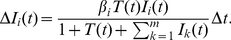
(2)This fraction undergoes mutation and is changed accordingly in the population. The mutation step is carried out by assuming that mutation occurs independently at every locus with probability 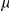.

In order to perform recombination, we assume at every time-point that (i) a fraction 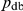 of all infected cells is doubly infected, (ii) amongst these doubly infected cells, the cells infected with two different strains 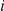 and 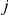 occur at a frequency 
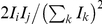
, which implies that the infection of a target cell by one virus strain happens independently from infection of the same target cell by another virus strain, (iii) a cell doubly infected with virus strains 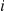 and 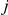 produces a new infection between the time 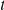 and 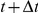 with the probability



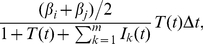
(3)(iv) in the course of such infection event, strains 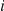 and 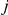 are enclosed in the same viral capsule with the probability of 50%, and that (v) the average number of crossover events is equal to 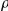 and crossover-sites are randomly distributed across the viral genome. Likewise, the newly recombined strains substitute the old strains in the population of infected cells.

### Measures

#### Escape types

We distinguish between two types of escape, E and EC. The escape E is the escape which only carries a mutation at the escape locus and regardless of the type of allele at the compensatory locus; the escape EC is the escape which carries a mutation at both escape locus and the compensatory locus (see Fig. 1C). Unless mentioned which type of escape mutation is analyzed, we refer to the E escape type.

#### Time of escape

The average time of escape of a mutant with 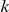 escape mutations is measured by calculating the total abundance of CD4+ cells infected with viruses with 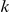 escape mutations and capturing the time when the number of these cells exceeds 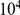.

#### Mean number of escapes

The mean number of escape variants in the population at a given time is calculated by the mean Hamming distance of the escape types from the wild type. For example, in order to calculate the mean number of E escape variants, we calculate the abundance of all strains with the number of E escapes equal to zero, one, two, etc., then multiply each by its Hamming distance from the wild type, and finally weight by the total virus load.

#### CTL avidity

Each CTL clonal sub-population has its assigned sensitivity (avidity) value 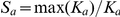, where 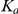 is the Michaelis-Menten constant assigned to each clone. Thus, the least sensitive population has mean avidity of 1. For each run (unless mentioned otherwise), we randomly choose the 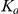 values between 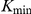 and 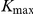.

#### CTL diversity

The diversity of the CTL population is a measure of their distribution. At each time point in one simulation run we calculated the diversity of the CTL response by the use of the Shannon diversity index 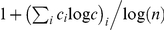 where 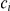 is a frequency of each CTL clone and 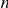 is the number of CTL clones. The mean diversity was calculated by first averaging over all simulations runs and then over time.

## Results

The aim of this study was to examine the impact of recombination on the immune escape of HIV. To that end, we considered a deterministic, population dynamic model of HIV infection with a stochastic implementation of mutation and recombination [33]–[35], and examined the viral-immune dynamics with and without recombination (see Methods). Using the model we quantified the impact of recombination on a number of important characteristics of the viral population (viral load, emergence of escape, diversity, etc.) as well as a number of important characteristics of the immune system (strength, distribution of CTL clones, etc.).

### Recombination accelerates the acquisition of escape mutations

**Figure 2:**
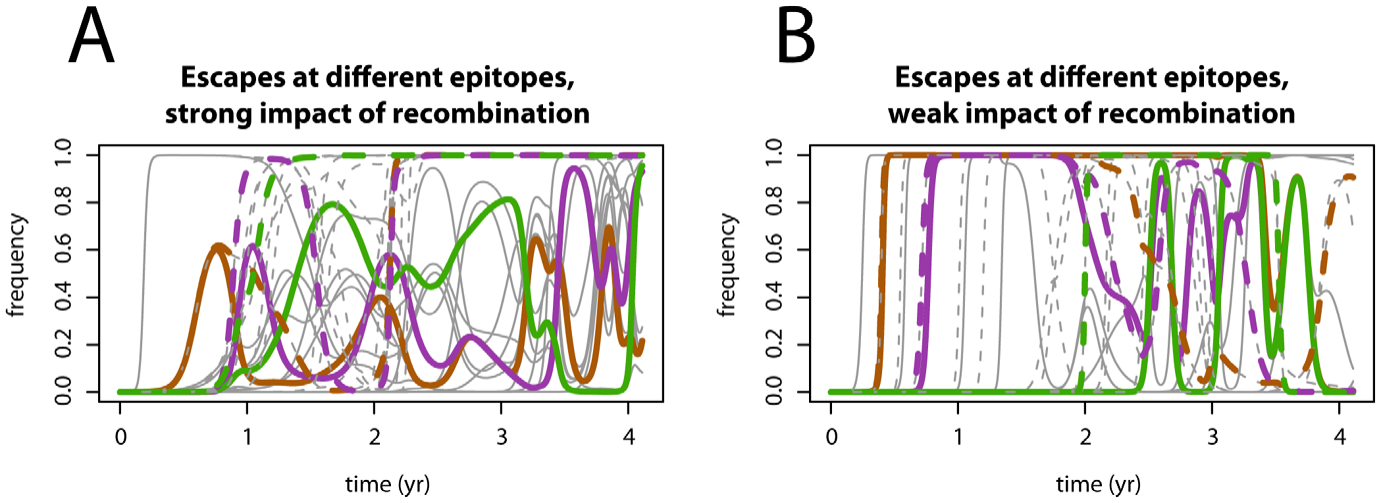
Example simulation runs of the virus escape dynamics. Simulation runs without recombination are shown with solid lines, and with recombination with dashed lines. Plotted are frequencies of escapes at all 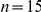 loci as a function of time, and in each case three chosen loci are printed in colour. Panel A shows an example simulation run for a parameter setting in which the impact of recombination is strong. Panel B shows an example simulation run for a parameter setting in which the impact of recombination is weak. Recombination mostly, but not always, accelerates the emergence and fixation of immune escapes. Parameters used in panel A: 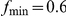, 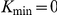 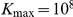, 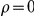. Parameters used in panel B: 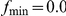, 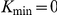 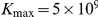 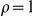; the remaining parameters are the same and are defined in Table 1.

A key question is whether recombination can accelerate the emergence of escape mutations in HIV, and if so, whether this is beneficial for the virus. Fig. 2 shows two representative simulation runs of the immune escape dynamics with and without recombination. The figure shows the frequencies of escape mutations at different epitope loci with three chosen escape frequencies shown in colour. Fig. 2A shows an example of dynamics where the impact of recombination is high. Fig. 2B shows an example of dynamics where the impact of recombination is low. This illustrates that recombination can, but does not generally accelerate the emergence and the fixation of immune escape. Furthermore, the distinct escape dynamics which can be seen in both panels suggest that the characteristics of the immune response play an important role in the scale of the recombination impact, as will become evident in the next section. (Even though the virus recombines with the same strength, the properties of not only the virus but also the immune system lead to a much weaker effect of recombination.)

**Figure 3:**
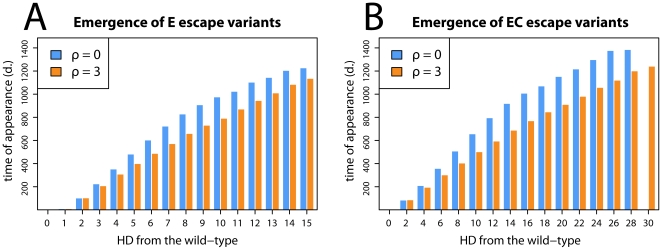
The average times of appearance of successive accumulations of escape mutations. In both panels the time of emergence of mutants with a given number of escape mutations is shown. Orange and blue bars correspond to runs with and without recombination, respectively. A shows the time of appearance of E escapes (escapes at the escape locus). Panel B shows the time of appearance of EC escapes (escapes at the escape and compensatory locus). The simulations show that recombination accelerates the emergence of escape mutations. Parameters used: 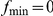, 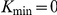 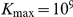; the others are defined in Table 1. The plot shows an average of 10000 simulation runs.

In order to assess a general impact of recombination on the evolution of escape in HIV, we measured the mean time of appearance of escape mutations during infection. In particular, we measured an average speed of accumulation of successive mutations from the time point of infection, i.e. the average time of appearance of a single mutant, the average time of appearance of a double mutant, etc. The results are presented in Fig. 3 where panel A shows the average times of appearance of successive E escape mutants and panel B shows the average times of appearance of successive EC escape mutants (see Methods and Fig. 1C for an explanation of E and EC escape mutants).

The figure shows that in runs with recombination successive escape variants appear faster than in the runs without recombination, and that the accumulating effect of recombination increases with the number of escape mutations. The impact of recombination is even stronger for EC than for E escape types. Since EC escapes need two point mutations, the results suggest that genetic shuffling not only helps bringing together escape mutations from different epitopes but also bringing together escape and compensatory mutations at the same epitope (see Discussion). Thus, our model shows that recombination accelerates the emergence of escape variants by clustering together various escape mutations in the virus.

**Figure 4:**
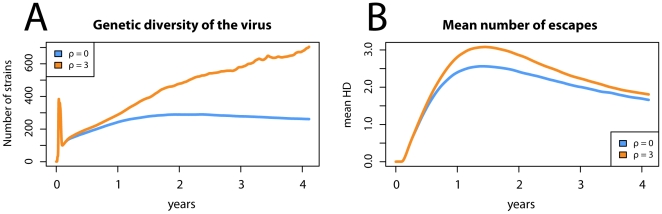
The effect of recombination on chosen parameters of the model. Orange and blue bars correspond to runs with and without recombination, respectively. Panel A shows the viral diversity as a number of strains in the population (results for other measures of diversity were qualitatively similar). It can be seen that recombination has a significant impact on diversity regardless of the stage of infection. Panel B shows the mean number of escapes measured by the mean HD at the escape loci. The panel shows that recombination accelerates the evolution of immune escape in the virus in the early phase of infection because it accelerates the acquisition of the escape mutations when they are beneficial. Note that the strain with mutations at all loci (HD = 30) has arisen only in the runs with recombination (one blue bar missing in panel B). The parameters are identical as in Fig. 3 and the plot also shows an average of 10000 simulation runs.

On the other hand, the acceleration of the emergence of escape mutations is likely to have other consequences on the virus dynamics, and thus we next examined which of the determinants of the viral population are significantly affected by genetic shuffling. We found that recombination has a considerable impact on viral diversity as well as the average number of escape mutations in the population. The results are shown in Fig. 4 where the average over many stochastic simulation runs is considered. Fig. 4A shows the viral genetic diversity as a function of time since the beginning of infection. One can see that due to the faster appearance of escape variants, a higher level of genetic diversity is achieved in virus populations with recombination. Fig. 4B shows the mean number of E escapes in the viral population as a function of time (see Methods). The runs with recombination resulted in an increased level of escape on the population scale early in the infection, however after 2–3 years the same runs (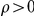) showed only a marginally higher number of escapes than the runs without recombination (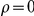). The initial increase in the number of escapes occurred because of a high selective pressure to escape the CTL responses, resulting in a subsequent decrease of the stimulation of T cells and hence of the selective pressure. At the same time, an increase in the mean number of escapes decreased the fitness of the virus population, making it susceptible to the invasion by strains with the wild-type allele (results not shown). As a result, the escape epitopes became selected against during the chronic stage of infection.

In line with this interpretation, we observed little or no reversion to the wild-type in simulations where the cost of escape was low or absent (results not shown). The higher mean number of escapes with 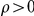 suggests that the process of selecting escape variants in the primary phase of infection was accelerated by the mechanism of recombination. Interestingly, we observed that recombination had no significant effect on the viral load unless the process of escape led to the appearance of an escape mutant with a substantially decreased kill rate and a relatively high replicative capacity (results not shown; see Discussion).

### CTL dynamics and the impact of recombination

Several measures of the T cell response are used extensively in immunological literature (e.g. immunodominance, breadth, strength). However, using them to characterize the CTL dynamics during a viral infection has the disadvantage that these measures not only result from the properties of the immune cells but are are strongly affected by the interactions of the immune response with the virus and the stochastic effects. In order to characterize the response itself, one could measure the avidity distribution of the T cell population for a given antigen, but such quantification in experimental assays remains not feasible. A mathematical model, on the other hand, has the advantage that the sensitivity of each CD8+ T cell clone to the wild-type virus is defined before the simulation run (by the avidity value 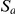; see Methods). Therefore, in this study we propose to quantify the CTL response by the mean avidity and the variance of avidity in each run. In this way, (a) two independent variables are used to quantify the immune response, while typical measures used in the literature can be read off the plot, and (b) a correspondence between the measures of the T cell population and the observed immune dynamics are investigated.

**Figure 5:**
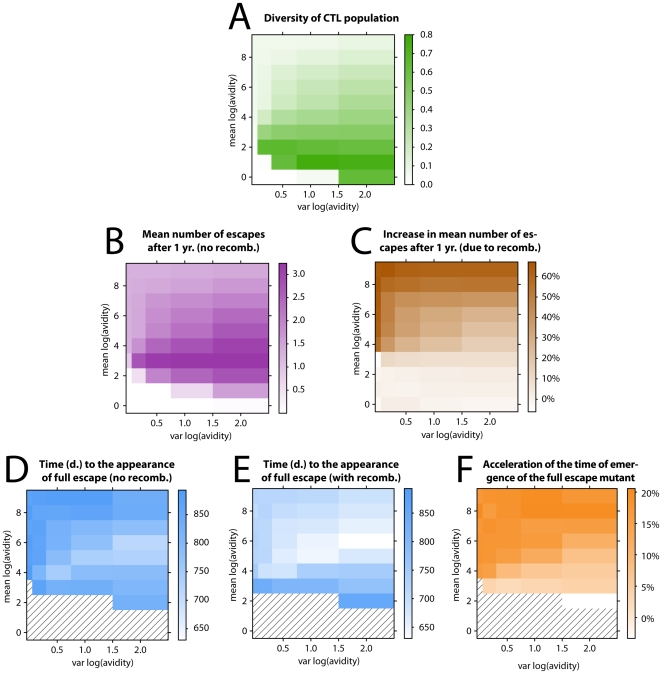
CTL dynamics and the impact of recombination. Figures are plotted as a function of mean avidity and variance of avidity of the CTL response. Panel A shows the mean diversity of the CTL population which decreases with increasing mean (except for an initial increase in avidity) and decreasing variance in avidity. Panel B shows the mean number of escapes during infection without recombination after 1 yr. of infection, which has an optimum for a given mean avidity of response and correlates well with the diversity of the CTL response in panel A. Panel C shows the factor of increase in the number of escapes after one year (cf. panel B) due to recombination, which is the strongest for broad and even responses. Panels D and E show the time of appearance (days) of the full escape variant without and with recombination, respectively. The hatched areas show runs in which the full escape mutant did not appear within the time of 1000 days. The impact of recombination on the time of emergence of the full escape mutant, shown in panel F, corresponds well to the impact of recombination on the number of escape mutations after one year, shown in panel C. The mean and variance of log avidities (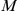 and 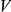, respectively) plotted in all panels are defined in each simulation, and the distribution of avidity values is drawn from a uniform distribution in each simulation run between 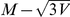 and 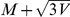. The parameters used: 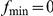, 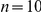, 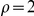. All other parameters are given in Table 1. Each point in the plot shows an average of 5000 simulation runs.

The results are displayed in Fig. 5 where panel A shows the diversity of the CTL response as a function of mean and variance of the T cell avidity (see Methods) averaged over many simulations (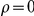 assumed but results for 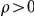 are qualitatively the same). As expected, the diversity of the CTL response decreases (responses become more even) as the variance of avidity decreases (low diversity for low avidity occurs because all clones are equally weak). However, increasing the mean avidity at a constant variance (with the exception of the zero variance and the very weak response) increases the evenness and hence the breadth of the response. Thus we see that the relation between the avidity and the breadth of the response can be non-trivial and counter-intuitive.

Fig. 5B shows the mean number of escapes at the escape loci after one year from infection, as a function of the mean and the variance of avidity. Fig. 5C shows the factor of increase in the measure in panel B due to recombination. One can see that as the potency (mean avidity) of the immune response increases, the efficiency of escape reaches an optimum, namely an initial increase in the impact of recombination is followed by a decrease. The initial increase occurs because with a higher avidity the strength of the immune system increases and so does the selective pressure. However, as explained above, by increasing the mean avidity the hierarchy of the immune response changes yielding a broader CTL response. Such immune response effectively targets the virus at many loci, disabling the evasion of the immune system (escape becomes too costly). However, at the same time that the immune response broadens, the impact of recombination becomes stronger. This is particularly evident for potent and non-immunodominant CTL responses, i.e. for runs with high mean and small variance in avidity. We see qualitatively the same pattern when measuring the acceleration of the appearance of the full escape mutant (Fig. 5D–F).

Why is the impact of recombination stronger for broader (non-immunodominant) responses? The reason is that when all effector responses are similarly strong, the expected time of escape at any epitope will be comparable, meaning that all epitopes will escape on average at a similar time because they will all become advantageous at a similar time. A multiple number of escape variants increasing in frequency will in turn allow for almost any combination of those mutations to be advantageous too, possibly outgrowing variants with a smaller number of escape epitopes. This creates an environment in which genetic shuffling will have a significant effect. By contrast, immunodominant responses will favour different escape variants at different times, which will decrease the diversity in the viral population, and hence the variance which recombination can act on. This is why we see a stronger impact of recombination on the number of escapes in models with little or no immunodominance.

We also examined the impact of recombination for a decreased cost of escape, and, in accordance with the intuition, we observed an increased effect of recombination on the emergence of escapes (results not shown). With decreasing cost of escape, the impact of recombination on the emergence of escape variants increased markedly. This is because fitter escape variants competed more efficiently with the wild-type variants which in turn, by increasing viral diversity, enhanced the impact of recombination. Finally, as we observed that a decreased level of immunodominance is generally associated with an increased breadth of the response and also with an increased number of CD8+ cells, we conclude that broader and stronger (higher activation and killing) immune responses are likely to increase the impact of viral recombination on the escape dynamics.

## Discussion

In this study we show that recombination in HIV accelerates the initial phase of viral coevolution with the immune system. We demonstrate that random shuffling of the genetic material (a) accelerates the emergence of escape mutations (both with and without compensatory mutations), (b) increases viral diversity during infection (in line with the experimental observations; see [41]), (c) accelerates the acquisition of escape mutations in the early stage of infection. Furthermore, our model exhibits a negative correlation between the impact of recombination and the efficiency of escaping the immune system. In particular, we observe that the impact of recombination is strongest in broad, non-immunodominant systems. Although our analysis focuses on the escape from cytotoxic T-lymphocytes, similar effect might occur with escape from other virus-specific responses including antibodies.

Generally, we see a higher impact of recombination with than without compensatory mutations, suggesting an acceleration in the adaptation of the virus due to recombination in spite of positive epistasis (a double mutant has higher fitness than a single mutant). This may seem paradoxical as under positive epistasis, recombination may be expected to decelerate adaptation [42]. We see three potential explanations for this paradox: (i) stochastic effects in this model may override the effects of possible epistasis [11]; (ii) the epistasis experienced by the virus is also influenced by the CTL dynamics, and these could therefore outweigh the positive epistasis of compensatory mutations with respect to reproductive fitness; and (iii) more generally the model here is substantially more complex than the population genetic models that have been used to determine the mentioned effects of epistasis on recombination, such that it is difficult to translate their observation to the situation considered here.

Depending on the total fitness cost of escape, and thus also on the number of escape loci, a mutant can arise that escapes at all loci. Because such full escape is no longer controlled by the immune response, the appearance of this mutant is accompanied by a substantial increase in viral load. The time of appearance of the full escape mutant depends strongly on the recombination rate. However, whether such total escape ever occurs in late stage patients is currently unclear.

Generally, we find that viral recombination increases the set-point viral load. Given the moderate magnitude of this increase (typically 5–10%, data not shown) and the large variance seen between clinical measurements of the virus load, it is not an effect that could easily be detected in clinical data even if there were considerable variation in recombination rates between different viral isolates. The fact that in our model recombination has only a weak effect on virus load, however, is compatible with strong selective pressures on escape mutants, as observed experimentally [23], [43]–[47], because large changes in viral fitness *in vivo* do not necessarily induce substantial changes in virus load [48], [49].

As common strains are likely to be targeted more strongly by the immune response, a negative frequency dependent selection may ensue, leading to potentially cyclical frequency dynamics between the virus and the immune system. This behaviour shows a resemblance with the so-called Red Queen dynamics in evolutionary biology, where fluctuating selection in host-parasite systems leads to allele frequency cycles and the maintenance of genetic variants over long periods of time. One of the leading hypothesis for the evolution of recombination (Red Queen Hypothesis) states that such circumstances can explain the evolutionary maintenance of genetic shuffling [50]–[52] (see [29] for a review). Here, we have focused on examining the consequences rather than the evolution of recombination rate. Nevertheless, the fact that viral recombination accelerates the acquisition of beneficial mutations in the virus indicates that recombination, like in the Red Queen scenario, could be advantageous for the virus. However, our model differs in a number of ways from the typical Red Queen models (multiple loci, absence of persistent allele fluctuations, interaction models, etc.), and whether the observed benefit of recombination stems from the similar effects as in the Red Queen Hypothesis is a topic that warrants further investigation.

Although the primary aim of this study has been the impact of recombination on the coevolution of HIV with the immune system, the results presented here might shed light on the evolutionary history of recombination in HIV (or other retroviruses like SIV). On the one hand, we have found that recombination can have a strong impact on the speed of acquisition of escape mutations, and thus arguably, on the adaptation of the virus to the new hosts. This suggests that the within-host selection acting on the virus might select for non-zero recombination rates. On the other hand, the total viral load has been correlated with the transmission probability of HIV, and since we have seen that recombination moderately increases the total virus load, the between-host selection acting on the viral recombination is likely to be weak or zero. This also emphasizes the importance of the within-host stage of evolution of HIV. However, what evolutionary forces act on recombination and how, as well as whether HIV had enough time to evolve its recombination rate, remains a matter of discussion.

HIV and the immune system remain one of the best qualitatively and quantitatively characterized coevolutionary systems. However, a better insight into the coevolutionary dynamics underlying the interaction of the virus with the immune system, as well as into the population dynamical consequences of recombination, requires an improved understanding of the parameter space defining these interactions. First, the recently underlined importance of macrophages in the development of HIV infection [53] as well as of an increased recombination rate in those cells [54] suggests that the inclusion of macrophages in a model of recombination might qualitatively affect the outcome of this study. Second, we have seen a positive correlation between the number of crossover events in HIV and the mean number of escape mutants in the late stage of infection (results not shown). Therefore, a better understanding of the relation between viral fitness *in vivo* and the number of escape mutations in the chronic phase of the infection might yield further insight into understanding the optimal recombination rate in HIV. Third, our results show that of particular importance is the estimation of the fitness of escape mutants in the context of the immune system (*in vivo*). The first component of this fitness is the cost that these mutations induce in the absence of the immune response, which could be measured by quantifying their effect on the replicative capacity of HIV *in vitro*. The other component of the fitness *in vivo* reflects the interaction of the virus and the immune response. This is encapsulated by the parameter avidity, which describes both the activation of the immune response by the virus as well as its subsequent recognition and the killing by the immune system. Obtaining more quantitative data on the specificity and diversity of the T cell repertoire recognizing the virus would allow better understanding of the effect of recombination on the population dynamics of the virus, and more generally would enable more quantitative approach to the causes underlying immunodominance.
